# Patterns of skeletal fractures in child abuse: systematic review

**DOI:** 10.1136/bmj.a1518

**Published:** 2008-10-02

**Authors:** Alison M Kemp, Frank Dunstan, Sara Harrison, Susan Morris, Mala Mann, Kim Rolfe, Shalini Datta, D Phillip Thomas, Jonathan R Sibert, Sabine Maguire

**Affiliations:** 1 Welsh Child Protection Systematic Review Group, Clinical Epidemiology Interdisciplinary Research Group, School of Medicine, Cardiff University, University Hospital of Wales Heath Park, Cardiff CF 2XX; 2 Radiology Department, Cardiff and Vale NHS Trust, School of Medicine, Cardiff University; 3 Support Unit for Research Evidence, Cardiff University; 4 Orthopaedic Department, Cardiff and Vale NHS Trust, School of Medicine, Cardiff University

## Abstract

**Objectives** To systematically review published studies to identify the characteristics that distinguish fractures in children resulting from abuse and those not resulting from abuse, and to calculate a probability of abuse for individual fracture types.

**Design** Systematic review.

**Data sources** All language literature search of Medline, Medline in Process, Embase, Assia, Caredata, Child Data, CINAHL, ISI Proceedings, Sciences Citation, Social Science Citation Index, SIGLE, Scopus, TRIP, and Social Care Online for original study articles, references, textbooks, and conference abstracts until May 2007.

**Study selection** Comparative studies of fracture at different bony sites, sustained in physical abuse and from other causes in children <18 years old were included. Review articles, expert opinion, postmortem studies, and studies in adults were excluded.

**Data extraction and synthesis** Each study had two independent reviews (three if disputed) by specialist reviewers including paediatricians, paediatric radiologists, orthopaedic surgeons, and named nurses in child protection. Each study was critically appraised by using data extraction sheets, critical appraisal forms, and evidence sheets based on NHS Centre for Reviews and Dissemination guidance. Meta-analysis was done where possible. A random effects model was fitted to account for the heterogeneity between studies.

**Results** In total, 32 studies were included. Fractures resulting from abuse were recorded throughout the skeletal system, most commonly in infants (<1 year) and toddlers (between 1 and 3 years old). Multiple fractures were more common in cases of abuse. Once major trauma was excluded, rib fractures had the highest probability for abuse (0.71, 95% confidence interval 0.42 to 0.91). The probability of abuse given a humeral fracture lay between 0.48 (0.06 to 0.94) and 0.54 (0.20 to 0.88), depending on the definition of abuse used. Analysis of fracture type showed that supracondylar humeral fractures were less likely to be inflicted. For femoral fractures, the probability was between 0.28 (0.15 to 0.44) and 0.43 (0.32 to 0.54), depending on the definition of abuse used, and the developmental stage of the child was an important discriminator. The probability for skull fractures was 0.30 (0.19 to 0.46); the most common fractures in abuse and non-abuse were linear fractures. Insufficient comparative studies were available to allow calculation of a probability of abuse for other fracture types.

**Conclusion** When infants and toddlers present with a fracture in the absence of a confirmed cause, physical abuse should be considered as a potential cause. No fracture, on its own, can distinguish an abusive from a non-abusive cause. During the assessment of individual fractures, the site, fracture type, and developmental stage of the child can help to determine the likelihood of abuse. The number of high quality comparative research studies in this field is limited, and further prospective epidemiology is indicated.

## Introduction

Skeletal fractures are diagnosed in up to a third of children who have been investigated for physical abuse.^1^ ^2^ ^3^ The fractures are often occult,^1^ ^4^ and they occur in infants and toddlers who cannot give a causal explanation. Children who have been physically abused represent a small proportion of the total number of childhood fractures. Most children who sustain fractures do so from falls, motor vehicle crashes, or other non-abusive trauma.^5^ In addition, a small group of children are more susceptible to fractures owing to underlying conditions that contribute to bone fragility.^6^ All health professionals who see children should be able to recognise the characteristics of fractures resulting from abuse and initiate child protection investigations where necessary, to prevent further injury that could be fatal.^7^ In reality, the possibility of child abuse is often overlooked in clinical practice.^8^ ^9^

We systematically reviewed the published world literature to answer the question “what features differentiate fractures resulting from abuse from those sustained from other causes?” We aimed to identify indicators that can help clinicians to identify cases of suspected child abuse when a child presents with a fracture for which the cause cannot be confirmed. We explore the strengths and limitations of the current evidence base and make recommendations for future research in this field. We anticipate that this review will enable the development of evidence based clinical guidelines. It will also contribute to the knowledge and understanding of the scientific evidence that expert medical witnesses are expected to have to support their opinion in the family and criminal courts.

## Methods

We did a literature search of international publications for original studies, using the databases and keywords listed in figure 1 and box 1. We included studies in all languages, as well as references from relevant studies, textbooks, and conference proceedings.

**Figure fig1:**
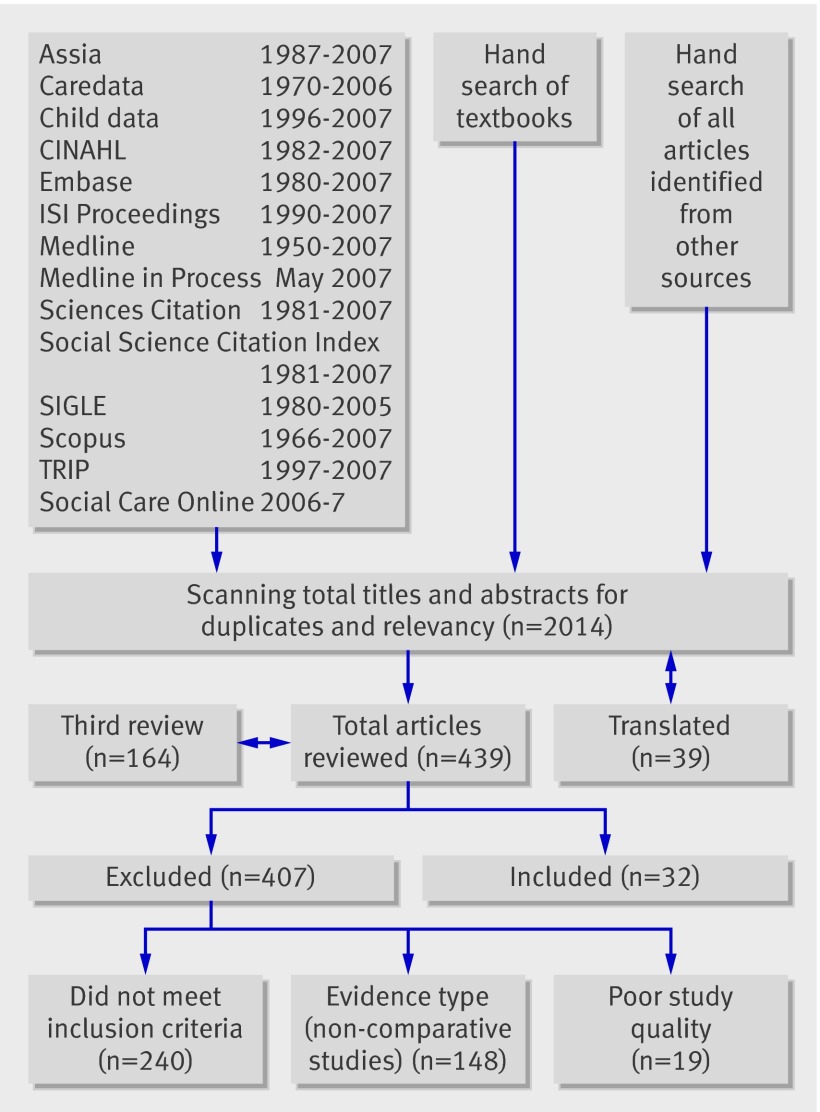
**Fig 1** Search strategy

**Box 1: Keywords and phrases used in database search**

1. child abuse.mp.
2. child protection.mp.
3. (battered child or shaken baby or battered baby).mp.
4. 1 or 2 or 3
5. (child: or infant: or baby).mp.
6. non-accidental injur:.mp.
7. non-accidental trauma.mp.
8. (non-accidental: and injur:).mp.
9. soft tissue injur:.mp.
10. physical abuse.mp.
11. (or/6-10) and 5
12. 4 or 11
13. fractur:.mp.
14. 12 and 13
15. (investigat: adj3 fract:).mp.
16. (radiolog: adj3 fractur:).mp.
17. (roentgen: adj3 fract:).mp.
18. skeletal survey.mp.
19. bone scan:.mp.
20. isotope bone scan:.mp.
21. radionuclide.mp.
22. scintigraphy.mp.
23. tomography, x-ray computed/
24. ((paediatric or pediatric) adj3 radiolog:).mp.
25. ((paediatric or pediatric) adj3 nuclear medicine).mp.
26. or/15-25
27. (ageing adj3 fractur:).mp.
28. ((dating or date) adj3 fractur:).mp.
29. (pattern: adj3 fractur:).mp.
30. healing.mp.
31. or/36-42
32. 26 or 31

We included comparative studies of children under 18 years old that described the distribution of fractures identified on radiographs, in which the fractures resulting from physical abuse were compared with those from other causes. We excluded review articles; expert opinion; consensus statements; studies of all age groups in which we could not separate data on children; studies judged to be methodologically weak owing to significant bias, confounding factors, case attrition, or incomplete ascertainment or in which the fracture pattern was the primary factor used to define abuse; and studies of outcome, management, or postmortem investigation.

Each study had two independent reviews (three if disputed) by specialist reviewers in the Welsh Child Protection Systematic Review Group, including paediatricians, paediatric radiologists, orthopaedic surgeons, and named nurses in child protection. We critically appraised each study by using data extraction sheets, critical appraisal, and data extraction forms based on 2001 guidance from the NHS Centre for Reviews and Dissemination.^10^

We classified included studies according to the child protection outcome decision and whether abuse had been excluded in the non-abused group (table 1). In the absence of a “gold standard” diagnostic test for child abuse, we used ranking schemes that were designed to ensure the best security of diagnosis.^11^ We judged studies against the highest standard (rank 1), where “a case outcome” of abuse had been made by a multi-agency child protection group or a legal panel using all available information relating to the case, a perpetrator had admitted abuse, or the abuse was witnessed. Lower ranked studies gave no details about how authors had come to a decision. In the non-abuse cases, the highest ranked studies had actively excluded the possibility of child abuse.

**Table 1 tbl1:** Ranking of definitions of child abuse and exclusion of abuse in comparison groups

Ranking	Criteria
**Definition of child abuse**	
1	Abuse confirmed at case conference or family, civil, or criminal court proceedings; admitted by perpetrator; or witnessed abuse
2	Abuse confirmed by stated criteria including multidisciplinary assessment
3	Abuse defined by stated criteria
4	Abuse stated but no supporting detail given
5	Suspected abuse
**Exclusion of abuse in non-abused group**	
1	Abuse actively excluded by stated criteria; witnessed traumatic cause; or confirmed organic cause
2	Exclusion of abuse implicit in case definition used or stated criteria given
3	Aetiology of non-abuse group merely stated
4	Aetiology of comparison not stated

We estimated the probability of abuse according to individual bony sites. We did a meta-analysis of cross sectional studies of consecutive cases of children with fractures seen in a given hospital or regional centre over a given time period.

Comparing these studies was not straightforward because many factors differed between them. Inclusion criteria varied; some studies excluded motor vehicle crashes, and others excluded pathological causes. For the purposes of our analysis we attempted to exclude motor vehicle crashes and fractures that occurred as a consequence of surgery, as we thought that these cases did not pose a clinical dilemma. The definition of abuse that was used to classify cases varied between studies. Some used a category of confirmed abuse, either excluding cases of suspected abuse or combining them with the non-abuse cases, whereas others combined confirmed and suspected abuse cases. Where data were sufficiently detailed, we accounted for this in our analysis. Age distribution varied greatly between studies, as did the site and type of fracture covered by the study.

For each bone for which the data justified meta-analysis, a forest plot shows the calculated probability of abuse with 95% confidence interval for all studies, plotted by year of publication. The estimates of the probability of abuse showed considerable heterogeneity between studies,^12^ so we fitted a random effects model.^13^ This method models heterogeneity by assuming that each study has a probability of abuse associated with it and that these form a probability distribution between studies. We estimated this probability distribution by a bayesian method, using WinBugs (Spiegelhalter), and derived a 95% credible interval to summarise the probability of abuse. The width of this interval reflects the degree of heterogeneity between studies as well as the number of children included in the studies.

In addition to the meta-analysis, we have provided a descriptive analysis about specific features that relate to each fracture site. These conclusions are all drawn from the comparative studies that met our inclusion criteria.

## Results

We included 32 comparative studies overall.^w1-w32^ We included 26 cross sectional studies in the meta-analyses; six additional studies provided useful comparative data but were not eligible for meta-analysis. Seventy eight per cent of studies were done in the United States; 78% had a ranking of 1-3 (table 1) for abuse, and 80% had a ranking of 1 or 2 for the non-abused group (web extra tables 2-6). All were retrospective studies and based in the hospital setting. However, studies variously included children attending or admitted to hospital. Data sources included reviews of a combination of medical records, social records, and radiographs. A small proportion implemented independent review of records or radiographs by several investigators who were blinded to case allocation. Web extra tables 2-6 summarise citations, results, and critical appraisals, according to the relevant fracture site.

### Fracture patterns in physical abuse and non-abuse

Seven studies compared the distribution of fractures in cases of abuse and non-abuse. Fractures resulting from abuse predominantly occurred in infant and toddler age groups. Worlock and colleagues studied children under 12 years old and showed that 80% of all fractures from abuse were seen in children under 18 months.^w1^ In contrast, 85% of fractures not caused by abuse occurred in children over 5 years. Figures from the study gave an estimated population annual incidence of fracture due to abuse of 4 per 10 000 children under 18 months. The authors estimated that in children under 18 months, one in nine fractures were due to confirmed abuse compared with one in 205 for those aged between 19 and 60 months and none in children over 5 years. Six further studies estimated that between 25% and 56% of fractures in children under 1 year of age arose from child abuse; prevalence figures varied between studies according to the definition of abuse and the inclusion criteria used.^w2-w7^ Studies showed that in children under 3 years old, skull fractures were by far the most common fracture type in both abused and non-abused children (web table 2).^w3 w4^

Worlock found a highly significant association between multiple fractures and abuse—for example, 74% (26/35) of abused children had two or more fractures compared with 16% (19/116) of non-abused children.^w1^ This was supported by Leventhal and colleagues in their study of fractures from traumatic causes but not by McClelland and Heiple’s study, in which half of the children in the non-abused group had factors predisposing to bone fragility (web table 2).^w4 w5^

### Fractures of lower limbs

Thirteen studies of femoral fractures met the criteria for meta-analysis.^w3 w6 w8-w18^ These included 1100 children under the age of 15, of whom 222 were classified as confirmed abuse, 120 were classified as suspected abuse, 223 had been involved in motor vehicle crashes or violent trauma, 29 had pathological fractures, and 506 were from other non-abusive incidents. Four of these studies looked specifically at fractures of the femoral shaft.^w10 w14-w16^

For the studies that included the combined categories of suspected and confirmed abuse, the overall estimated probability of abuse given a femoral fracture was 0.43 (95% confidence interval 0.32 to 0.54) (top panel, fig 2), excluding children who were involved in a motor vehicle crash or violent trauma. When we excluded cases of suspected abuse, the probability that a femoral fracture was due to confirmed abuse was 0.28 (0.15 to 0.44) (bottom panel, fig 2).

**Figure fig2:**
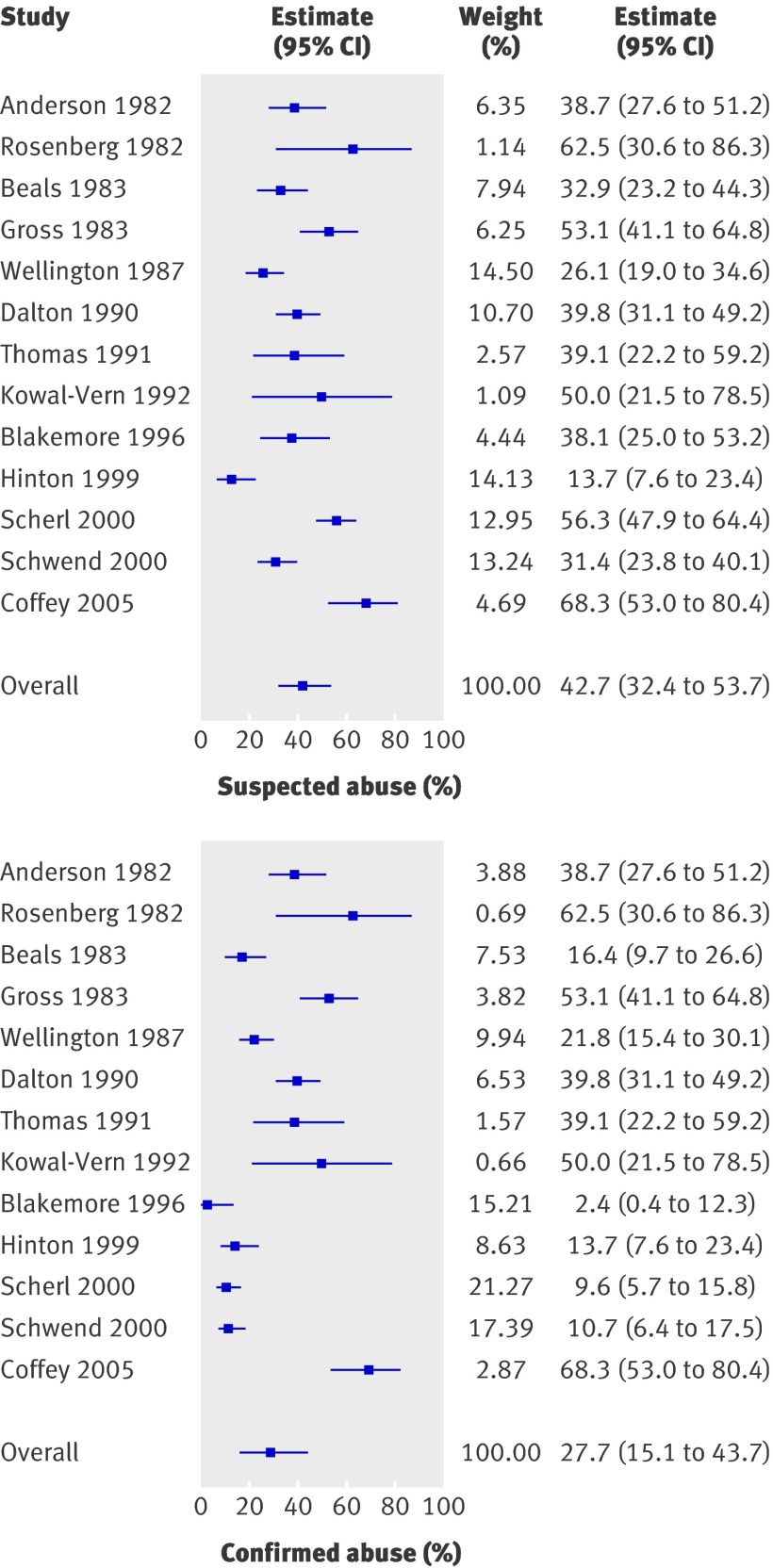
**Fig 2** Probability of abuse given femoral fracture after exclusion of children involved in motor vehicle crash or violent trauma, using threshold of suspected and confirmed abuse (top) and threshold of confirmed abuse (bottom)

Five studies provided sufficient data to enable a comparison between the mean ages of children who had a femoral fracture from abuse and those who had femoral fractures from other causes.^w8-w10 w15 w16^ However, in some cases we had to estimate standard deviations; we deliberately overestimated these, to give conservative results. In these five studies, the mean age in the abused cases was significantly less than in the non-abused ones. Schwend and colleagues looked at motor milestones and found that fractures from abuse were significantly more common in children who were not walking (web table 3).^w16^

The most common location of femoral fracture in both abused and non-abused children was the mid-shaft of the femur.^w9 w10^ Overall, we found no difference in the distribution of transverse, spiral, or oblique fractures between the groups.^w6 w8 w10 w12 w15 w17^ Only one study analysed spiral fractures by age; it found that a spiral fracture was the most common abusive femoral fracture in children under 15 months, and no significant difference existed between the distribution of spiral fractures in abuse and non-abuse in children older than 15 months.^w12^ Metaphyseal fractures were reported in a greater proportion of abused than non-abused children (web table 3),^w8 w9^ but insufficient data were available for further meaningful analysis.

Only two studies described tibial or fibular fractures.^w3 w11^ In children under 3 years old, Kowal-Vern and colleagues reported one fracture from abuse out of a total of eight fractures. For children under 18 months, Coffey and colleagues stated that 96% (23/24) of all tibial or fibular fractures resulted from abuse.

### Fractures of upper limbs

Six cross sectional studies looked at abusive humeral fractures: two studies examined specific fracture types,^w19 w20^ and four studies were suitable for meta-analysis.^w3 w6 w17 w21^ These studies included a total of 154 children who sustained a fracture of the humerus, of whom 30 were classified as abused, 23 had suspected abuse, 100 had fractures resulting from non-abusive injury, and one was involved in a motor vehicle crash. All children were under 3 years old.

The overall estimate of the probability of suspected abuse, given a humeral fracture, in a child under 3 was 0.54 (0.20 to 0.88) (top panel, fig 3). When we excluded cases of suspected abuse, the probability that a humeral fracture was due to abuse was 0.48 (0.06 to 0.94) (bottom panel, fig 3).

**Figure fig3:**
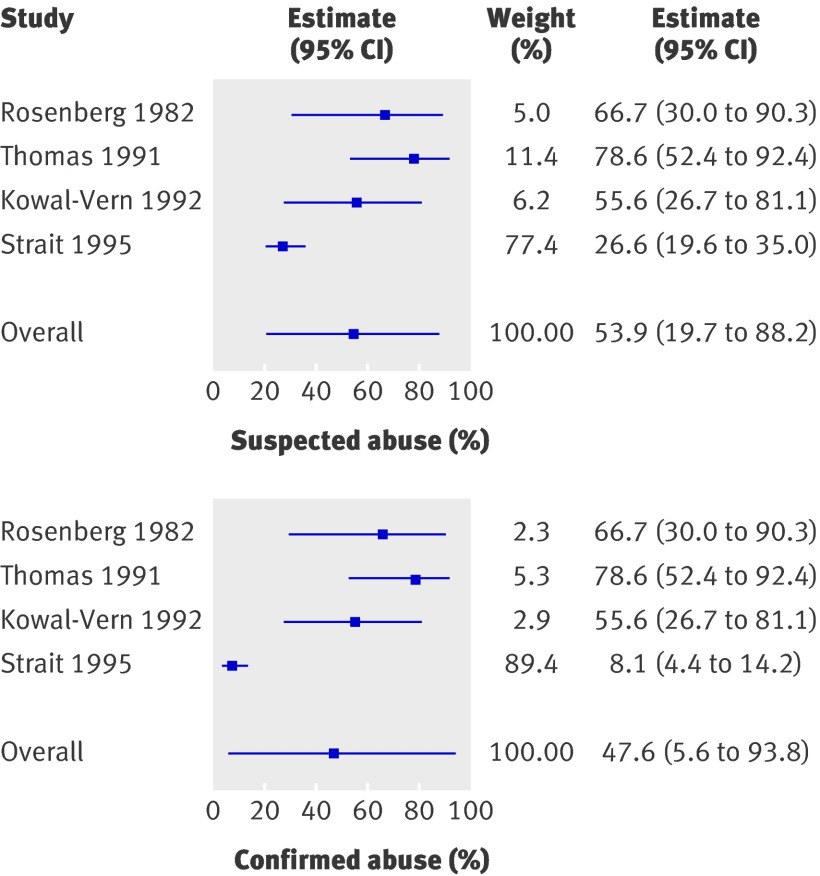
**Fig 3** Probability of abuse given humeral fracture, using threshold of suspected and confirmed abuse (top) and threshold of confirmed abuse (bottom)

Strait and colleagues gave the lowest probability for abuse.^w21^ This study adopted very high diagnostic criteria for abuse and excluded cases of abuse that were diagnosed before the discovery of the humeral fracture (web table 4). The authors analysed the data by age and found that the prevalence of abuse was significantly greater in children under 15 months with a humeral fracture than in those between 15 months and 3 years of age. Shaw and colleagues confirmed this finding in their analysis of fractures of the humeral shaft.^w20^

Supracondylar fractures were more likely to be associated with non-abusive injury.^w17 w21^ This was confirmed in a large cross sectional study that looked specifically at displaced supracondylar fractures in 388 children of all ages.^w19^ Seventy nine per cent of these fractures occurred after a fall, and only 0.5% were the result of abuse. However Strait and colleagues reported supracondylar fractures from abuse in three of 10 abused children under 3, and the authors cautioned that a supracondylar fracture should not be assumed to have non-abusive causes without careful consideration.^w21^ The most common type of humeral fracture from abuse in children under 15 months of age was a spiral/oblique fracture (web table 4).^w1 w20 w21^

The study of Kowal-Vern and colleagues was the only one to comment on the proportion of radial and ulnar fractures that were caused by abuse.^w3^ They identified an overall abuse rate of 25% (4/12). Worlock and colleagues described the location of fractures in 10 children with radial/ulnar fractures from abuse, of which two were greenstick, one was transverse, one was periosteal, and three were metaphyseal chip fractures; in comparison, 37/40 fractures from other causes were greenstick fractures.^w1^

### Rib fractures

Seven cross sectional studies including rib fractures were suitable for meta-analysis.^w22-w28^ They included details of a total of 233 children: 128 were abused, 24 had diagnosed bone dysplasia, 17 were preterm babies with perinatal complications, 43 had fractures due to motor vehicle crashes or violent trauma, seven had postsurgical fractures, three had birth injuries, and 11 had fractures from unknown or other non-abusive traumatic causes. After exclusion of children who were involved in a motor vehicle crash, documented violent trauma cases, and postsurgical cases, the pooled estimate of the probability of abuse given a rib fracture was 0.71 (0.42 to 0.91) (fig 4). The data presented did not allow us to estimate a probability for confirmed cases. Five studies included conditions that predispose to bone fragility as a possible cause and showed that osteopenia of prematurity or bone dysplasia were common causes of rib fractures in the infant/toddler population.^w22-w24 w27 w28^

**Figure fig4:**
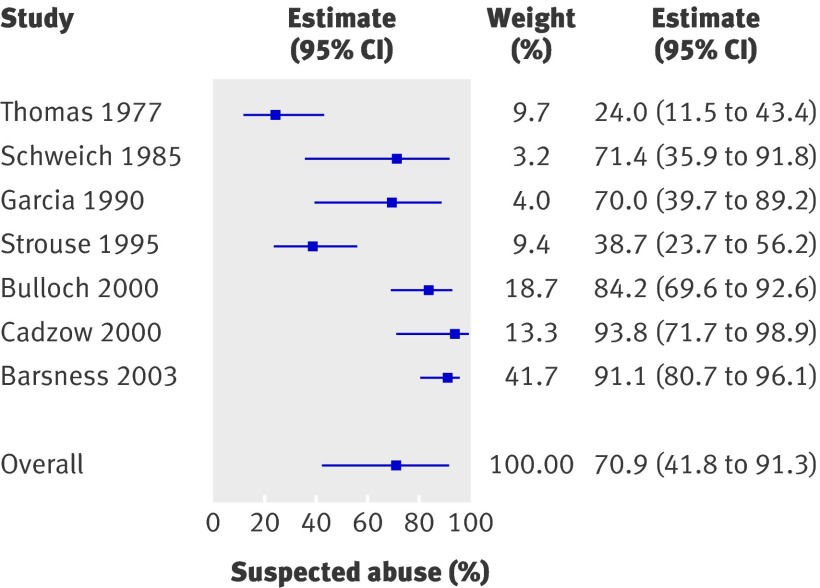
**Fig 4** Probability of abuse given rib fracture after exclusion of children involved in motor vehicle crash or violent trauma, using threshold of confirmed abuse

The radiological investigations varied between studies and may explain the variation in prevalence figures. One study included oblique views of the chest in the investigation of children with suspected abuse,^w22^ and one study included additional scintigraphy in half of the abused group.^w24^ Both of these investigations increase the sensitivity for detection of rib fractures. The details of chest radiograph technique were not reported in the remaining studies. Two studies included an independent radiology review,^w22 w23^ whereas the remainder relied on a review of case and radiograph records.

All but one study showed that children who had rib fractures from abuse had more rib fractures than those who had not been abused (web table 5).^w23^ Rib fractures from abuse were reported at any location on the rib^w23-w25 w28^; they could be unilateral or bilateral. Two studies confirmed that anterior fractures were significantly more common in abuse and that lateral fractures were more common in non-abused children.^w22 w24^ Findings on posterior fractures were variable. Barsness and colleagues found that posterior rib fractures were significantly more common in abuse than in non-abuse,^w22^ whereas neither Cadzow and Armstrong nor Bulloch and colleagues supported this finding.^w23 w24^

### Skull fractures

Seven studies of children with skull fractures met our criteria for meta-analysis.^w3 w5 w6 w29-w32^ These involved a total of 520 children under the age of 6.5 years; 124 were classified as abused, 18 had fractures caused by motor vehicle crashes or violent trauma, and 378 were non-abused. All but one study covered an infant/toddler age group.^w32^

Skull fractures are more commonly reported after non-abusive trauma than after abusive head injury; the point estimate of the probability of abuse given a skull fracture was 0.30 (0.19 to 0.46) (fig 5). We could not give an estimate for confirmed cases of abuse alone or exclude motor vehicle crashes.

**Figure fig5:**
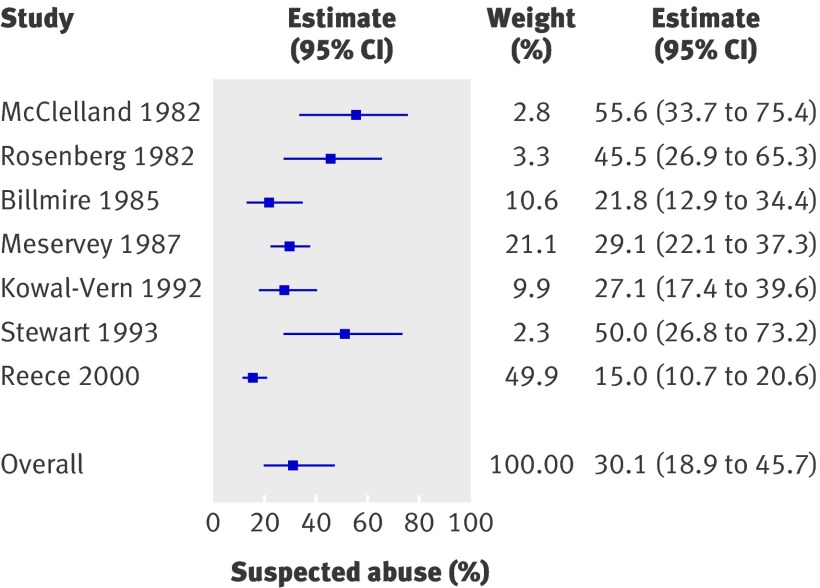
**Fig 5** Probability of abuse given skull fracture, using threshold of suspected and confirmed abuse

The most common fracture site in both the abuse and non-abuse groups was parietal,^w30^ and the most common fracture type was linear.^w31^ This finding was supported in two further comparative studies that were not eligible for the meta-analysis.^w1 w4^

The significance of complex fractures varied between studies (web table 6). Meservy and colleagues found no significant differences between the two groups with respect to depressed, diastatic, or complex fractures but found that multiple or bilateral fractures or those that crossed suture lines were significantly more common in abused children.^w30^ Billmire and Myers found that depressed skull fractures were equally common in abuse and non-abuse; complex multiple fractures were associated with intracranial injury in 4/30 abused infants but in none of the 54 non-abused cases.^w29^ Stewart and colleagues studied children under 3 months of age and stated that diastatic and multiple fractures were more common in abuse than in non-abuse.^w32^ However, neither Reece and Sege nor Leventhal and colleagues found any significant difference between the prevalence of complicated fractures in the two groups.^w4 w31^

## Discussion

This systematic review has combined cross sectional studies in a meta-analysis to estimate the probability of abuse given rib fractures, skull fractures, or long bone fractures. We have also identified features related to the child or type of fracture sustained that should alert clinicians to consider physical abuse as a possible cause (box 2).

**Box 2: Features associated with possible child abuse**

Physical abuse should be considered in the differential diagnosis when an infant (under 18 months) presents with a fracture in the absence of an overt history of important trauma or a known medical condition that predisposes to bone fragility. The following indicators can be used to inform decisions about the likelihood of child abuse:

- Multiple fractures are more common after physical abuse than after non-abusive traumatic injury
- A child with multiple rib fractures has a 7 in 10 chance of having been abused
- Multiple rib fractures are more common in abuse than in non-abuse
- A child with a femoral fractures has a 1 in 3-4 chance of having been abused
- Femoral fractures resulting from abuse are more commonly seen in children who are not yet walking
- A child aged under 3 with a humeral fracture has a 1 in 2 chance of having been abused
- Mid-shaft fractures of the humerus are more common in abuse than in non-abuse, whereas supracondylar fractures are more likely to have non-abusive causes
- An infant or toddler with a skull fractures has a 1 in 3 chance of having been abused
- Parietal and linear skull fractures are the most common type of skull fracture seen in abuse and non-abuse
- No clear difference exists in the distribution of complex skull fractures between the two groups

In the absence of a confirmed traumatic cause, rib fractures had the highest probability (71%) of being caused by abuse. Humeral fractures had a one in two chance of being the result of abuse, and for femoral or skull fractures the estimated probability was one in three. Fractures resulting from abuse have been described in virtually every bone in the body. Published studies of bone fractures not described in this review were limited to small case series and case studies, and a comparative analysis was not possible.^14^

### Causes of fractures

We identified a strong inverse relation between the age of the child and the likelihood of a fracture from abuse. A greater breakdown of age related data presented in the primary studies would have been beneficial to the analysis. Overall, the primary studies confirmed that the pattern and causes of fractures varied considerably at different stages of the child’s age and development. Osteopenia of prematurity and bone dysplasia were reported in the infant and toddler age group and should be considered as part of the differential diagnosis, with appropriate investigations to exclude associated clinical, skeletal, and biochemical abnormalities.^6^ The prevalence of non-abusive traumatic causes increased with age. This is unsurprising because of the different hazards and environments that children are exposed to as they grow up. Clinicians who work in the child protection field need an understanding of some of the basic biomechanics behind childhood injuries to inform their decisions as to whether causal explanations for a fracture are plausible.

### Limitations of review

During this study, we faced many of the difficulties identified by researchers who do diagnostic systematic reviews, for which the methods have yet to be fully developed.^15^ The quality of any review of this nature is heavily dependent on good observational studies that are well reported and have been done in a consistent manner. However, a high degree of heterogeneity existed between studies, which is reflected in the forest plots. The between study variation that is seen may be due to factors such as the different age ranges of the children included in studies and the exact site of the fractures covered, but it may also be due to variation in the actual frequency of abuse. Unfortunately, the data available in the primary studies were insufficient to allow any further investigation of this.

The ranking of abuse varied considerably across studies; we attempted to account for this in the analysis, but identifying the different thresholds for suspicion of abuse that were applied was difficult. International definitions and thresholds for abuse vary greatly. The vast majority of studies were from the United States, where definitions of abuse differ from state to state; although these research findings are valuable, they are not always directly applicable to the United Kingdom.

All of the studies were retrospective and may have been compromised by incomplete datasets. They had the benefit, however, of information derived from case investigations and outcomes. The absence of detail on the radiological techniques used in many of the larger studies weakens the data, particularly when comparing the numbers of fractures seen in abuse and non-abuse. The optimal investigation strategy to identify all fractures in children with suspected abuse includes a skeletal survey including oblique views of the chest, which has a much higher sensitivity for identifying rib fractures than a standard chest radiograph.^16^ ^17^ We often could not determine what investigations children with suspected abuse had received. Most non-abused children are unlikely to have skeletal surveys if these are not clinically indicated. Fractures reported in these groups are those that are clinically evident, and occult fractures may be under-represented. As a result, an intrinsic and unavoidable bias exists within these studies.

No comparative data were available to determine the probability of abuse for many fracture sites. This was particularly evident for metaphyseal fractures. Historically, metaphyseal fractures have been thought of as strong predictors of abuse,^18^ but we could not find comparative studies to support or refute this hypothesis. The most comprehensive studies of metaphyseal fractures in physical abuse have been published by Kleinman and colleagues.^19^ ^20^ ^21^ ^22^ These rely on a comparison of radiological findings and an analysis of the histopathology of metaphyseal lesions in a group of fatally abused children. The studies did not meet our inclusion criteria.

### Fracture specific indicators

We have produced a descriptive analysis from comparative studies to identify additional indicators of abuse for certain fractures. For fractures of the femoral shaft, a key discriminator was the motor developmental level of the child.^w16^ Clinicians should consider physical abuse in a child with a femoral shaft fracture who is not yet walking. Unexplained mid-shaft fractures of the humerus should be carefully assessed to exclude child abuse in children under 15 months, whereas supracondylar fractures are strong predictors of non-abusive trauma, particularly in older children. When skull fractures were considered, the type of fracture sustained was not discriminatory. In 1984 an influential study of severely abused children stated that depressed diastatic, growing, and multiple fractures were more common in abuse than in non-abuse.^23^ The study included a considerable number of fatally abused children and was not eligible for inclusion in this systematic review. This review did not confirm these associations, with the possible exception of multiple fractures, bilateral fractures, or fractures that crossed the suture lines.^w30^ Similarly, data on the location of rib fractures did not confirm the widely held belief that posterior fractures are discriminators for abuse. Multiple rib fractures in any location, in the absence of overt trauma, were strongly associated with abusive injury.

### Future directions for research

Published research in this field has its limitations, and high quality prospective studies would be valuable to examine the deficiencies that we have identified. Studies need to focus on preschool children and draw on large populations to derive a meaningful sample size. They should collect standardised data and radiological investigations at the point of presentation and relate them to the outcome of the case. Researchers must ensure a high security of diagnosis of abuse, adequate investigation of cases of suspected abuse for possible organic causes, and exclusion of abuse in non-abuse groups. Studies must include disabled children, who are significantly under-represented in the literature but are known to be at high risk of abuse.^24^ Published studies to date have evaluated the association between a single characteristic or a limited number of characteristics of a fracture with abuse or non-abuse. However, we know that making an assessment of the likelihood of child abuse relies on clinicians evaluating the whole picture and fitting several pieces of information into a “jigsaw” to determine whether the child has been abused. Future research should attempt to reflect this by including a multivariate analysis of features and how they fit together.

### Conclusions

We have done a thorough systematic review of the international literature on fractures and child abuse. The main benefits of this work are threefold. Firstly, we have identified that a high proportion of fractures in infants arise from physical child abuse. We recommend that in the absence of an overt cause child abuse should be considered and investigated as part of the differential diagnosis in this age group. However, no fracture on its own can be used to diagnose child abuse. We have shown that the age and motor developmental level of the child together with the type and site of the fracture are important features to consider when assessing these children.

Secondly, we have provided a comprehensive literature review with detailed critical appraisal of the best comparative studies that are currently available.^14^ We hope that this will help clinicians who are acting as expert medical witnesses in court and child protection proceedings to appreciate the benefits and limitations of the evidence base that they are using to inform opinion. We hope that this will prevent conflicting medical evidence in the court setting, thereby assisting everyone involved with these complex cases. Finally, and most importantly, we have identified many deficiencies in the scientific research in this field, and we have identified methodological limitations that we hope will inform high quality research in this field in the future.

**What is already known on this topic**

Children who have been physically abused often sustain bony fractures
Different fracture types have variously been described as having a high probability for abuse

**What this study adds**

No one fracture in isolation is specific for physical abuse
Rib fractures, regardless of type, are highly specific for abuse in the absence of an overt traumatic or organic cause
Fractures from child abuse are significantly more common in children under 18 months of age than in older children, which should inform the differential diagnostic approach in this age group

## Notes

Contributors: AMK is the research programme director, was responsible for methodology and data analysis, and did reviews. FD did the statistical analysis. SD, SH, and SM were the main radiology reviewers, checked and agreed the radiology detail of the review quality standards, and were involved in data analysis and writing the manuscript. MM did literature searches. PT was the orthopaedic adviser to the project, did reviews, and checked the orthopaedic content of the paper. JRS acted as project co-director, reviewer, and paediatric adviser to the project. SM was the principal investigator, did reviews, scanned studies for relevance, compiled the appendix, and analysed data. KR contributed editorial work on the manuscript and management of the review process, provided methodological data on the review process, and compiled website content at www.core-info.cf.ac.uk. AMK is the guarantor.

Review team: M Barber, P Barnes, M Bhal, A Butler, J Bowen, R Brooks, S Datta, B Ellaway, R Frost, C Graham, S Harrison, M James-Ellison, N John, A Kemp, A Maddocks, S Maguire, S Morris, A Mott, A Naughton, C Norton, H Payne, L Price, I Prosser, J Sibert, P Thomas, E Webb, C Woolley.

Ethical approval: Not needed.

Provenance and peer review: Not commissioned; externally peer reviewed.

## Notes

**Cite this as: ***BMJ* 2008;337:a1518
